# The Potential of Antibody Technology and Silver Nanoparticles for Enhancing Photodynamic Therapy for Melanoma

**DOI:** 10.3390/biomedicines10092158

**Published:** 2022-09-01

**Authors:** Zaria Malindi, Stefan Barth, Heidi Abrahamse

**Affiliations:** 1 Laser Research Centre, Faculty of Health Sciences, University of Johannesburg, 55 Beit Street, Doornfontein, Johannesburg 2028, South Africa; 2 Medical Biotechnology and Immunotherapy Research Unit, Department of Integrative Biomedical Sciences, Institute of Infectious Disease and Molecular Medicine, Faculty of Health Sciences, University of Cape Town, Anzio Road Observatory, Cape Town 7925, South Africa

**Keywords:** melanoma, photodynamic therapy, SNAP-tag, nanoparticles, antibody, CSPG4, zinc phthalocyanine

## Abstract

Melanoma is highly aggressive and is known to be efficient at resisting drug-induced apoptotic signals. Resection is currently the gold standard for melanoma management, but it only offers local control of the early stage of the disease. Metastatic melanoma is prone to recurrence, and has a poor prognosis and treatment response. Thus, the need for advanced theranostic alternatives is evident. Photodynamic therapy has been increasingly studied for melanoma treatment; however, it relies on passive drug accumulation, leading to off-target effects. Nanoparticles enhance drug biodistribution, uptake and intra-tumoural concentration and can be functionalised with monoclonal antibodies that offer selective biorecognition. Antibody–drug conjugates reduce passive drug accumulation and off-target effects. Nonetheless, one limitation of monoclonal antibodies and antibody–drug conjugates is their lack of versatility, given cancer’s heterogeneity. Monoclonal antibodies suffer several additional limitations that make recombinant antibody fragments more desirable. SNAP-tag is a modified version of the human DNA-repair enzyme, O6-alkylguanine-DNA alkyltransferase. It reacts in an autocatalytic and covalent manner with benzylguanine-modified substrates, providing a simple protein labelling system. SNAP-tag can be genetically fused with antibody fragments, creating fusion proteins that can be easily labelled with benzylguanine-modified payloads for site-directed delivery. This review aims to highlight the benefits and limitations of the abovementioned approaches and to outline how their combination could enhance photodynamic therapy for melanoma.

## 1. Melanoma

Melanoma is highly aggressive and has innate chemoresistance mechanisms [1]. As such, it is the most threatening skin cancer. Fewer than 5% of skin cancer cases are melanoma, but melanoma is the cause of over 80% of skin cancer-related deaths [2]. Currently, the standard of care for melanoma is resection; however, for surgery to be effective, the cancer must be detected early, and only local disease control is possible. If melanoma is only detected in the later stages, the risk of recurrence is high, and adequate treatment options are very limited, especially once the cancer has progressed to the metastatic phase, where chemoresistance is more likely [3].

Ultraviolet (UV) radiation over-exposure and gene mutations—most frequently in BRAF^V600E^, followed by N-RAS—are the most common causes of melanoma [4]. Aberrant mitogen-activated protein kinase pathway activation is the predominant pathogenic signalling pathway in melanoma and is usually activated as a result of BRAF and RAS mutations [5]. In this pathway, which has been well-described in the literature, increases in cell proliferation are observed, leading to nevi formation; as the tumour enters the radial growth phase (RGP), immortalisation and further proliferation occur; in the vertical growth phase (VGP), migration pathways are subsequently activated, leading to cell invasion into the deeper layers of the skin, and epithelial-to-mesenchymal transition begins; and in the final phase—the metastatic phase—metastasis and angiogenesis occur [6]. One of the other significant pathways that has been identified as contributing to melanoma progression is the NFκB pathway, which has been shown to promote tumour proliferation, survival and metastasis, leading to melanoma progression and increased metastatic potential [7]. The NF-κB complex protein is a transcriptional factor for multiple genes across various pathways. It plays three key roles of significance in melanoma progression: (1) it influences proinflammatory responses, including immune, inflammatory and acute phase responses, (2) it is an anti-apoptotic factor that facilitates the expression of anti-apoptotic proteins, such as Bcl-XL, tumor necrosis factor receptor-associated factors and inhibitor-of-apoptosis proteins, and (3) it increases cyclin D1, thus promoting cell growth [8]. Studies have demonstrated the overexpression of NF-κB family members in pre-cancerous nevi and in malignant melanoma cells. NF-κB has been shown to interact with BRAF to increase melanoma cell survival, and NF-κB activation is reportedly caused by deregulations in the RAS/RAF, PI3K/Akt and NIK signalling pathways. The result in transformed melanoma cells is increased proliferation and resistance to apoptosis [8,9]. Moreover, NF-κB inhibition has been shown to suppress melanoma growth and metastasis; thus, NF-κB has consequently gained interest as a therapeutic target for melanoma [10].

ATP-binding cassette (ABC) transporters are one mechanism by which melanoma cells can become drug resistant. ABC transporters are transmembrane proteins that facilitate cytotoxin efflux from the cells, limiting intracellular drug accumulation and bioavailability [11,12,13,14]. Additionally, while all cancers produce reactive oxygen species (ROS)—inherently subjecting their cells to oxidative stress conditions—and have well-developed antioxidant systems, the antioxidant capabilities of melanoma are superior. Melanocytes naturally contain various antioxidant substances that protect the skin from free radicals and oxidative stress [15,16]. Melanin precursors put melanocytes under continuous oxidative stress as they act as potent oxidants, but the melanosomes localise these substances, preventing damage to the cells. Melanin itself, in contrast, acts as a potent antioxidant, scavenging any free radicals. Thus, the melanosomes serve to sequester cytotoxic products from the cell and hold them in this membrane-bound organelle. This function is evidently useful for removing toxins from healthy skin cells. However, this process is proposed to facilitate chemoresistance by mopping up chemotherapeutic agents and preventing their action in melanoma cells, enabling therapeutic resistant, metastasis and recurrence with heightened tumorigenic power [17]. The American Society of Clinical Oncology [18] reported that patients in whom melanoma is detected early and who undergo resection at the primary tumour site have a 5-year survival rate of 99%. This rate drops to 68% once melanoma has invaded the lymph nodes, and to just 30% in the late metastatic stages. As such, it is evident that we need to formulate new therapies that are able to overcome the resistant nature of melanoma.

## 2. Photodynamic Therapy

**Figure 1:**
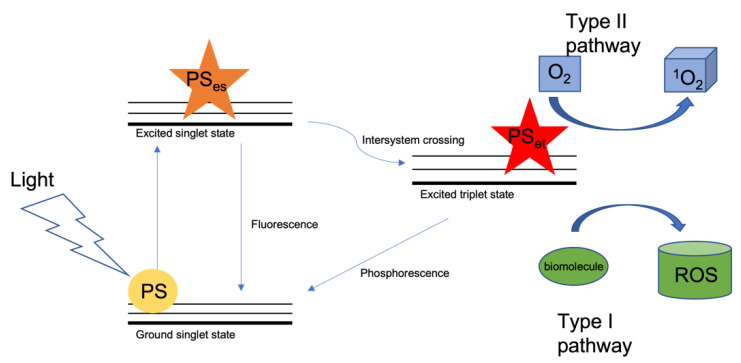
Schematic of photodynamic therapy. When light of a particular wavelength interacts with the PS, the PS goes from a ground singlet state to an excited singlet state. Thereafter, intersystem crossing occurs, and the PS enters an excited triplet state. It then interacts with (1) O_2_ to form ^1^O_2_ and (2) biomolecules to form ROS. Some excited singlet state PS molecules will return to their ground state, releasing fluorescence; others will return from the excited triplet state to the ground state, releasing phosphorescence.

Photodynamic therapy (PDT) is a treatment currently being investigated for melanoma, as well as other cancers and skin diseases. Briefly, in PDT, a photosensitizer (PS) is irradiated in the presence of molecular oxygen. This produces singlet oxygen (^1^O_2_) and ROS that are cytotoxic to target cells, inducing cell death through apoptosis, necrosis, autophagy and immunogenic cell death [19,20,21,22]. A PS is inert in its ground state, but upon activation with light of a wavelength within its absorption spectrum, it enters an excited singlet state. This state is very unstable, and when the PS decays, it fluoresces upon returning to its ground state, allowing for visualization of the PS that can be used for photodiagnosis [23]. The PS can alternatively undergo intersystem crossing and enter an excited triplet state that is much more stable, lasts longer and in which its electrons enter a higher-energy orbital [24]. It can then either decay to its ground state, emitting phorsphorescence, or, because this stable excited state allows time for energy transfer, it can interact with nearby biomolecules, bringing about cell death through: (a) the type I photooxidative pathway in which electrons or hydrogen ions are transferred to neighbouring biomolecules, which then trigger free radical reactions react by interacting with oxygen to produce various ROS, such as peroxides, superoxide ions and hydroxyl radicals, or (b) the type II pathway, in which ^1^O_2_ is created when the PS returns to the ground state and the released energy is transferred to ground state oxygen (Figure 1) [25,26,27]. So far, promising results have been shown for PDT in several non-malignant dermatological conditions and in some malignant skin cancers [28,29,30]; however, while PDT has been approved for the treatment of several cancers [31], it has yet to receive approval in melanoma. Table 1 lists all PSs that have been approved for cancer management.

An extract of the St John’s Wort (*Hypericum perforatum* L.) plant called hypericin is a PS that has shown therapeutic potential in melanoma. It absorbs UVA (315–400 nm) and visible light (548–593 nm) and has shown promise for eliminating cancerous skin cells in vitro [14,35,36]. Studies on this compound have demonstrated that apoptotic cell death is predominant for unpigmented melanoma cells, while necrotic cell death dominates in pigmented melanoma cells. This supports the fact that melanin is able to absorb UV-vis light, providing photoprotection from ROS production and associated apoptosis. Nonetheless, in pigmented melanomas, hypericin has been shown to localise in the melanosomes, suggesting that when the melanosome’s membrane is damaged upon irradiation, melanin precursors can leak into the cytoplasm and cause necrosis as they are cytotoxic outside of the melanosomes [36,37]. Hypericin has, however, been shown to interact with cytochrome P450 [38,39] and impact the pharmacokinetics and pharmacodynamic dynamics of drugs being taken concurrently [40], which therapeutically limits its utility in vivo.

While there are reports of successful in vitro outcomes for hypericin, the use of UV-vis PSs, such as hypericin, is arguably limited. Melanoma has an aggressive VGP [41] and as such, light-based treatments need to penetrate beyond the superficial layers of the skin, deeper into the dermis. However, the depth of light penetration is limited with UV-vis light because the absorption spectra of biomolecules such haemoglobin lie within the visible light range and are particularly high at wavelengths below 600 nm [42,43]. This overlap indicates that the UV-vis light will be absorbed and scattered in the biological tissue, which could restrict PS photoactivation and decrease PDT efficiency [44]. Furthermore, the melanin pigment offers photoprotection against harmful UV radiation as it is able to scatter and absorb UV radiation [2], and this too could optically limit the efficiency of PDT in pigmented melanomas. Therefore, treatment with UV-vis PSs might offer only sub-curative treatment, requiring a higher dosing frequency; unfortunately, prolonged or repeated UVA exposure can cause photo-aging, photo-hypersensitivity, immunosuppression and carcinogenesis [45,46].

Near-infrared (NIR) fluorescent dyes have recently piqued the interest of PDT researchers because they might be beneficial over conventional UV-vis compounds for several reasons. NIR light exhibits less absorbance and scattering, combined with superior tissue penetration in vivo because biomolecules such as oxyhaemoglobin, deoxyhaemoglobin and water absorb minimal light in this range and therefore interfere less with PS photoabsorption and resultantly produce better signal-to-noise ratios [42,47,48]. This is due to what is referred to as the “first optical window”, which refers to the NIR region from 650 nm to 950 nm at which the absorption coefficients for biological tissues are known to be minimal compared with those at visible light wavelengths, allowing for greater tissue penetration of NIR light [48,49,50]. Furthermore, many NIR dyes have higher extinction coefficients, which means that the NIR agents produce a higher phototoxic effect per photon absorbed compared with conventional agents, and thus fewer molecules of NIR PSs are needed to elicit comparable effects to those of UV-vis PSs. That is to say, NIR PSs are more efficient photoabsorbers. In addition, studies using NIR dyes, such as IRDye 700dx (IR700), have indicated that, unlike with UV-vis dyes that have side effects associated with prolonged exposure or ionizing radiation therapies such as those that utilize gamma rays or x-rays, repeated NIR light exposure is unharmful because NIR irradiation is not dose limiting [51,52]. Therefore, NIR PDT can better cater to long-term melanoma management and encourage eradication [53,54,55].

Although PDT is gaining attention, it depends on passive PS accumulation through the enhance permeability and retention (EPR) effect. It lacks targeted specificity; thus, the ‘scatter gun effect’ can lead to off-target effects in healthy tissue [56].

## 3. Nanoparticles

Nanoparticles (NPs) are organic or inorganic structures that are able to refine site-specific drug delivery by enhancing the biodistribution, uptake and release of drugs that are surface-immobilised on or encapsulated within them [57]. Their hydrophilicity improves drug solubility and, thus, pharmacokinetic capabilities [58]; they can be easily functionalised (e.g., to increase biocompatibility—and, thus, circulation times [59,60]—or with targeting ligands to achieve site-specific drug delivery) [61]; their small size facilitates intracellular tumour accumulation via the EPR effect; and they go unchecked by the immune system because they mimic biomolecules, allowing them to avoid interference [62,63]. Additionally, NPs exhibit large area-to-volume ratios (aspect ratio), allowing them to carry high drug loads, which reduces the minimal effective dose [64,65]. Some common organic NP formats include liposomes, dendrimers and polymers, while common inorganic NPs include metals (such as gold and silver), metal oxides (such as zinc oxide and iron oxide) and mesoporous silica [66]. While each has its benefits, inorganic NPs can be easily designed to possess optimal size, shape and optical properties [59,64].

By loading PSs into various NP formats, researchers have demonstrated the intensification of PDT effects in vitro. By using these drug carriers to deliver a PS, the uptake and intracellular concentration of the PS can be improved, as NPs improve solubility and distribution and facilitate passive diffusion across the cell’s lipid membrane [67]. Many NP formats have been studied for their favourable optical properties and physiochemical properties, such as biocompatibility, thermal stability, aspect ratio and surface functionalization [68,69].

Silver (Ag) NPs are known to be non-toxic and biocompatible and to possess antimicrobial and anti-inflammatory properties [68,69,70]. It has, further, been shown that AgNPs themselves have antitumour effects when used as PSs in PDT [71]. Mfouo-Tynga et al. [68] demonstrated this ability of AgNPs to decrease cancer cell viability and induce cell death in both breast and lung cancer cells, and Erdogan et al. [72] reported similar results in breast cancer cells, showing that AgNPs suppressed cell proliferation after PDT. The use of AgNPs in conjugation with tumoricidal drugs has also been reported, although this technique has not been well researched thus far. For example, Srinivasan et al. [73] showed that multifunctional PDT treatment with doxorubicin-conjugated AgNPs enhanced cytotoxicity to cancer cells compared with drugs alone. Aiello et al. [74] report that HeLa cervical cancer cells underwent cell death after coincubation and irradiation with pectin-coated AgNPs and a PS called riboflavin, although the NPs were not in this case used as a nanocarrier for the PS. Mahajan et al. [75] modified AgNPs with porphyrin for the photodynamic imaging of melanoma cells; however, the study did not focus on the tumoricidal ability of the conjugate. Nonetheless, they showed that the conjugate produced an enhance ^1^O_2_ quantum yield compared with free PS with regards to fluorescence. Liu et al. [76,77] conjugated a NIR PS to AgNP/carbon dot nanohybrids and found that the conjugate induced great levels of ^1^O_2_ and enhanced cell death in vivo in xenograft mouse models of breast cancer, compared with free PS or the nanohybrids alone. While several researchers have shown the photodynamic effect of AgNPs as PSs and the synergetic effects of this combined chemotherapy [77], there is little literature on the use of PS-loaded AgNPs being used in PDT.

El-Hussein et al. [78] have shown evidence that while gold nanoparticles (AuNPs) are being extensively studied for use in PDT [79], AgNPs exhibit greater photodynamic effect than do AuNPs. It has also been suggested that AgNPs might in fact be superior to AuNPs due to their higher extinction coefficients, ratio of scattering to extinction and field enhancement [80]. Moreover, while AuNPs reportedly exhibit photothermal effects during PDT [81], AgNPs have been shown to generate cytotoxic ROS following irradiation, which can augment the photodynamic effect of therapies using PSs [82,83].

NPs evidently have great value in drug delivery for cancer. However, while they have a preferential affinity for cancerous tissues, like PSs, they too depend on passive targeting though the EPR effect to enter cancer cells. This mean that, like PSs, they too might have a sub-optimal uptake into the target tissue and could be improved through functionalisation with targeting ligands.

## 4. Targeted Delivery

### 4.1. Monoclonal Antibodies and Antibody–Drug Conjugates

The fairly recent discovery of tumour-associated antigens (TAAs) and tumour-specific antigens (TSA) afforded us an entirely new understanding of cancer that has guided novel diagnostic and therapeutic strategies that utilise the biorecognition abilities of monoclonal antibodies (mAbs) to achieve site-mediated targeting of cancer cells. Melanoma, like all cancers, is a widely heterogeneous disease; each subtype, and even each individual tumour, can display a distinct gene signature, manifesting as countless unique antigen expression profiles [84]. Thus, different mAbs are required to target specific cancers. Antibody-based immunotherapies enable us to use antibodies to direct antitumoural activity to particular cellular targets, either exploiting the mAb’s immune activity or by delivering cytotoxic payloads as antibody–drug conjugates (ADCs) [85]. Immunotherapy thus offers a solution to the scattergun effect observed in PDT. Phototoxic agents can be selectively delivered to melanoma cells with limited off-target delivery and systemic side effects.

ADCs offer several notable advantages over naked mAbs as several factors are considered to potentially hamper successful cancer destruction using mAbs. First, mAb efficacy depends on effective receptor inhibition to induce an antibody-mediated immune response, whereby antibody-dependent cellular cytotoxicity and immune responses or complement-dependent cytotoxicity and cell lysis are activated via the fragment crystallizable (Fc) region, resulting in cell death [86,87]. However, these functions might not be reliable when acting on an immune system that is compromised and not functioning at full capacity [88,89]. Second, cancer cells can develop advanced immune evasion mechanisms and manipulate the surrounding microenvironment to facilitate immune tolerance [90,91], so mAbs need to work against this. Third, there is evidence that repeated mAb exposure causes cancer cells to respond adaptively by downregulating the target receptor, resulting in resistance [88,92]. Therefore, ADCs have gained popularity because an mAb carrying a drug molecule has a greatly increases chance of successfully causing toxicity to the cell. This means lower ADC doses are required compared with mAbs to elicit comparable responses, and ADC resistance will consequently develop suitably later compared with an mAb alone. Overall, ADCs allow us to widen the therapeutic window and make long-term cancer management a possibility [93].

Conventional cancer therapies use free toxins to kill transformed cells that are dividing aberrantly. However, they act non-specifically, including on healthy tissues, contributing to systemic toxicity. A myriad of dose-limiting side effects are experienced by patients on these regimes, and their quality of life is often drastically reduced, which influences treatment discontinuation [94]. Site-directed drug delivery using antibody technology can significantly reduce the toxic effects to non-cancerous cells, and this specificity enables us to use drugs that would be too toxic in the body if in free circulation, such as Auristatin F. Moreover, photosensitive compounds offer controlled drug activation. Even if some degree of off-target accumulation occurrs, without light at a particular wavelength, no effects will be seen at these sites [25]. Cleavable linkers, such as polyethylene glycol (PEG), also offer controlled drug release by preventing the release of toxins into the bloodstream and only allowing detachment from the antibody after cellular internalisation and exposure to the endolysosome’s acidic environment [95,96]. As we can see, ADCs provide additional control mechanisms to prevent undesirable, off-target cytotoxicity.

Apart from the cytotoxic properties of PSs, their innate fluorescent properties allow us to use the same molecule to not only treat cancer non-invasively but also image a tumour, track intracellular accumulation in real time during treatment and monitor treatment-induced changes (e.g., size, location and relative receptor expression) [23,97]. Conventional diagnostic techniques, such as biopsy and morphological examination, are invasive, and they do not offer a holistic assessment of the tumour; they tell us about pathological changes that usually only provide late-stage measures of tumour progression, including gross morphology and size changes [98]. Antibody-conjugated PSs offer a dual diagnostic and imaging modality, known as theranostics. Conventional diagnostic techniques also come with a certain risk of false-negatives and -positives, resulting in a) late-stage diagnosis and poor prognosis or b) unnecessary treatment of patients with non-malignant tumours [99].

The selectivity of antibody technology means that the unique cell surface expression signature of an individual patient can be screened, and the appropriate therapy with the highest likelihood of success can subsequently be administered, personalising the therapy.

### 4.2. Silver Nanobioconjugates in PDT

While there has been notable research on the conjugation of AuNPs to antibodies for the targeted PDT of cancer [61,100,101,102,103,104], there is limited literature on the similar functionalisation of AgNPs, despite the potential benefits reportedly offered by the latter. Tai et al. [105] functionalised AgNPs with anti-HER2 antibodies; however, they only investigated the imaging properties of this conjugate in mouse bladder carcinoma cells. Khristunova et al. [106] created voltammetric immunoassays to determine tick-borne encephalitis virus antibodies using AgNPs. However, in this case, the AgNPs were used as a detection label. Similarly, Szymanski and Porter [107] used antibody-conjugated AgNPs to develop an electrochemical immunoassay. Pollok et al. [108] investigated the use of cross-linkers to modulate mAbs–AgNP conjugation in terms of orientation of attachment, number of conjugations and antibody activity. Again, however, this study did not investigate therapeutic effect. Finally, Nima et al. [109] investigated the targeted delivery of doxorubicin-loaded Ag-decorated AuNPs in breast cancer and prostate carcinoma. They modified the NPs with anti-EpCAM antibodies and found that lower conjugate doses were required compared with free drug to elicit comparable levels of cell death. In this study, Ag was considered mostly for its detection properties.

As it stands, there is scope to further investigate the utility of AgNPs in antibody-mediated nano-PDT that combines the phototoxic activity of PDT with the antigen-targeting precision of immunotherapy and the enhanced drug delivery benefits of nanomedicine to create an sophisticated active targeting PDT strategy.

### 4.3. ADC Limitations

Though ADCs are being introduced enthusiastically in cancer therapy [110,111,112], one limitation in applied clinical settings is their current lack of versatility and personalisation, especially when considering the remarkable inter- and intra-tumour heterogeneity of cancer, which has an effect on drug resistance, treatment response and overall therapeutic outcome [113]. The FDA has approved only four mAb treatments for melanoma to date: namely, ipilumimab, which targets CTLA4; nivolumab and pembrolizumab, which target PD-1; and opdualag, a combination therapy including nivolumab and relatlimab-rmbw which targets lymphocyte activation gene-3. However, they have not approved any PDT treatments for melanoma [114,115,116,117]. This paucity of targeting agents leaves patients who do not respond to these drugs with no alternatives.

**Figure 2:**
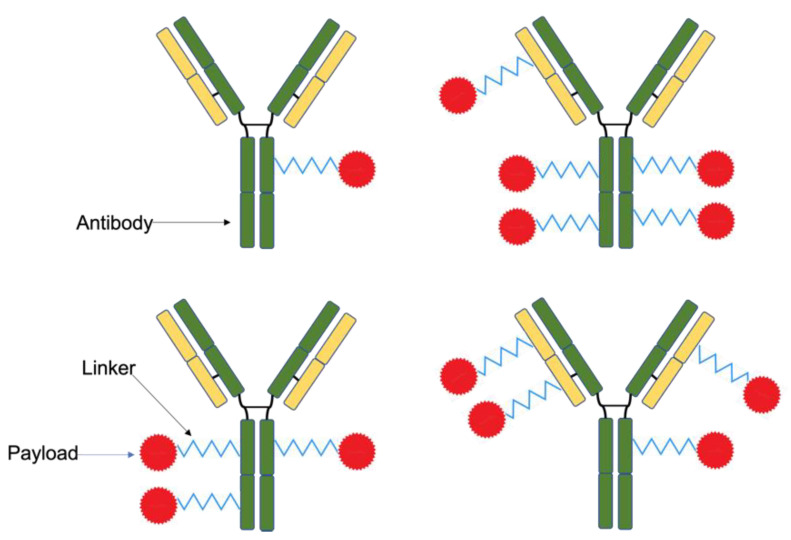
Heterogenous ADCs produced using conventional conjugation methods. Linking between the payload molecule and the antibody can happen across any of the available residues. Therefore, the drug-to-antibody ratio and the ADC configuration can differ. It is difficult to control this using conventional conjugation methods, and the properties of the conjugates vary as a result.

Further limitations include the difficulty in executing and controlling drug conjugation to mAbs. ADCs are conventionally formed by cross-linking the amino acid side chains of the antibody lysine residue with the payload, which can lead to heterogeneous conjugates because it is difficult to control where and to which exact side chain the linking occurs (Figure 2). As a result, the configuration and drug-to-antibody ratio (DAR) of each conjugate differs, which impacts their pharmacokinetic properties. Furthermore, batches are difficult to replicate using this method; thus, the efficacy and safety profile are difficult to determine [118]. If the DAR exceeds four, the conjugate might be identified by the body as a damaged protein and rapidly cleared from circulation. Therefore, drug load must be carefully considered when designing ADCs [112,119,120,121]. Another consideration during the conjugation process is how to preserve the antibodies’ structural integrity and biological functioning, which can be negatively impacted in the conjugation environment where pH, toxicity and temperature changes occur; furthermore, the drugs themselves can also impact the antibodies. If the antibody suffers any changes, this can affect its antigen binding region and, consequently, limit the specificity of its biorecognition [122]. Lastly, the first generation of mAbs and ADCs were developed from murine sources, which can cause immunogenicity [123]. Considering the abovementioned limitations, several solutions have been developed to enhance the ADC preparation process, e.g., the use of antibody fragments and recombinant antibodies.

## 5. SNAP-TAG Technology

**Figure 3:**
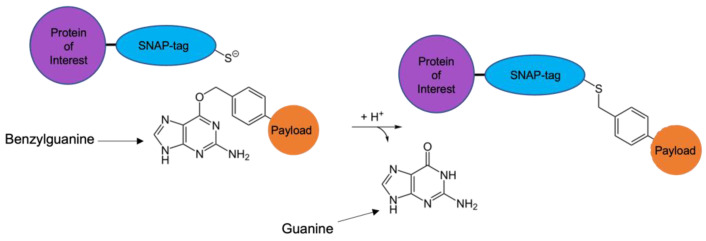
Autocatalytic reaction between SNAP-tag and BG-modified substrate. The benzyl alkyl group of the BG bonds covalently with the reactive cysteine residue on the SNAP-tag, forming a thioether; guanine is released in the reaction. The SNAP-tag fusion protein is now labelled with the payload molecule in a known DAR (1 to 1) and with a homogenous configuration.

One area of interest currently being studied to circumvent issues associated with current generation therapeutic antibodies is SNAP-tag technology, modified from the human DNA repair enzyme O^6^-Alkylguanine-DNA alkyltransferase (hAGT). hAGT functions by removing DNA alkyl adducts from the O^6^ position of guanine and transferring the alkyl group to its Cys145 reactive cysteine residues to release guanine. Benzylguanine (BG) functions as a substrate for hAGT. When exposed to BG, a covalent bond is formed between the two molecules; this causes hAGT to become irreversibly inactivated [124,125]. SNAP-tag is an engineered derivative of hAGT. The reaction between SNAP-tag and any BG-modified substrate is autocatalytic, covalent and 1:1 in stoichiometry, with the benzyl alkyl group of BG being transferred to the reactive cysteine residue on SNAP-tag to produce a stable thioether, releasing guanine (Figure 3). The coupling occurs at a distinct binding site, and the end product is homogeneous [126,127]. Moreover, because SNAP-tag is a derivative of a human protein, humanisation is not required and it does not carry the threat of immunogenicity, making it suitable for in vivo application in humans [128].

In order to achieve site-mediated action with SNAP-tag, the protein can be genetically fused to any desired targeting ligand. For example, by generating a fusion protein comprising SNAP-tag and an antibody single-chain variable fragment (scFv), a recombinant antibody is generated that can target the antigen of interest. The scFv is one of the smallest functional antibody formats in which the antibody’s antigen-binding properties are retained; however, these fragments are typically sensitive to direct chemical processing [129,130]. By generating a SNAP-tag-based fusion protein in which SNAP-tag acts as the site of chemical modification, we can avoid any potential harm to the ligand [131]. In contrast to traditional mAb-based ADCs, SNAP-tag reacts readily with BG and does not require additional conjugation steps or chemicals and catalysts, allowing for a greatly simplified protein labelling process.

The scFv-SNAP-tag vehicle has an optimal size (SNAP-tag, 19.4 kDa; scFv-SNAP-tag, ~50 kDa) to facilitate enhanced tissue penetrability, greater uptake and higher tumour-to-background ratio compared with full-length mAbs. In in vivo mouse imaging studies, it has shown to accumulate rapidly in the tumour soon after injection, followed by rapid renal clearance of excess vehicle [132,133]. The rapid clearance of excess vehicle from the bloodstream, combined with sufficient tumour retention, minimises background interference during visualisation and the risk of off-target cytotoxic effects. By comparison, full-length antibodies typically have more prolonged tumour retention, but they are cleared inefficiently due to their large hydrodynamic radius (≥5 nm) [125,134]. To reduce renal clearance and prolong the half-life of therapeutic proteins, PEGylation is known to improve the pharmacokinetics of therapeutic proteins and can be used to enhance drug availability and efficacy [135,136].

SNAP-tag technology provides simple solutions to the abovementioned concerns associated with mAbs and ADCs. Recombinant SNAP-tag antibodies are protected from harmful chemical alterations during conjugation, preserving the specificity and efficacy of the conjugate. Proof of concept for SNAP-tag-based photoimmunotherapy was provided by Hussain et al. [137] in 2011. They reported using the scFv-425 anti-EGFR fragment to create the scFv-425-SNAP fusion protein, which they conjugated to chlorin e6 and demonstrated successful eradication of EGFR-positive epithelial cancer cells. Since then, interest in SNAP-tag has been on the rise, with a few groups investigating its use in photoimmunotherapy across multiple cancers [97,138,139,140].

## 6. TAA Target Selection

There a several factors to consider when choosing an appropriate therapeutic marker for antibody-based therapies. First, it is imperative to select a TSA that is a neoantigen particular to the individual tumour or a TAA that is differentially expressed on the cancer cells vs. other tissues in the body. This ensures that the payload will be delivered selectively to the target cells and not cause toxicity to other, healthy cells. The target receptor must also be one that undergoes little to no secretion into circulation, otherwise antibody–antigen binding could occur in the bloodstream, causing the therapeutic to be used up, limiting uptake in the target cells and resulting in off-target effects [141].

Currently, the four FDA-approved immunotherapies for melanoma (nivolumab and pembrolizumab, which target PD-1; ipilimumab, which targets CTLA-4; and opdualag, which targets PD-1 and LAG-3 [114,115,116,117,142,143]) all function as immune checkpoint inhibitors, counteracting the immunosuppressive effects of T cell exhaustion in the tumour microenvironment [144,145,146,147]. However, there are currently no FDA-approved ADCs for melanoma [148].

One other potential ADC target for melanoma that is gaining interest is chondroitin sulphate proteoglycan 4 (CSPG4) [149,150]. CSPG4 is reportedly over-expressed >70% of melanomas [150]. It is a key role-player in cell survival and proliferation (affecting the RGP); cell adhesion, motility, and invasion (affecting the VGP); and migration and angiogenesis (affecting metastasis) [151,152,153]. High CSPG4 expression is observed in various immature and developing tissues when tissues are undergoing major reorganisation and cell motility is correspondingly high, and it exhibits restricted expression in some adult tissues; however, it is typically post-translationally down-regulated in normal adult tissues that are terminally differentiated [154,155,156,157]. Its over-expression in malignant cells is evidence of its contribution to tumour progression, and its differential expression in melanoma vs. healthy cells enables the selective targeting of melanoma. CSPG4 is also a permanently membrane-bound protein, thus it experiences limited secretion into circulation, making it an ideal target for antibody-based therapies [149,158]. Other viable targets that are currently under investigation for melanoma ADC treatment include melanotransferrin [159], c-Kit [160], HLA-DR [161], HER2 [162], transmembrane glycoprotein NMB [163] and AXL [164]. Table 2 provides a list of some PSs that are currently under investigation for use in melanoma PDT, indicating the use of NPs and/or antibodies.

## 7. Remaining Challenges

While PDT offers evident promise for the treatment of malignant melanoma, some challenges must be noted regarding the light delivery to metastases in less accessible areas of the body. Frontal diffusers have been used in clinical PDT settings to deliver light to superficial tumours (i.e., those less than 1 cm below the surface of the skin); however, needle catheters have been used to deliver light via a cylindrical diffuser to interstitial tumours (i.e., more than 1 cm below the skin). In the latter, multiple needle catheters are needed to ensure full tumour coverage, spacing needs to be considered and placement needs to image-guided, e.g., using ultrasound [55]. Endoscopy-assisted PDT using a frontal diffuser has also been reported for nasopharyngeal squamous cell carcinoma [172], as well as in the treatment of endoscopic ultrasound-guided PDT of pancreatic cancer using a needle catheter and cylindrical diffuser [173,174]. Nonetheless, metastases may be located in regions that are inaccessible even by endoscopy or needle catheter. As such, combination therapies, e.g., PDT and chemotherapy, might offer the solution to targeting not only primary tumours that can be reached through irradiation of the tissues, but also metastases located in less accessible areas in the body [30]. Biteghe et al. [14] reported a study in which they developed a chemoresistant melanoma cell line, resistant to dacarbazine, and subsequently tested the cytotoxic efficacy of a dacarbazine hypericin-PDT combination therapy. They found that while the chemoresistant cell line exhibited a decreased responses to dacarbazine, hypericin-PDT and combination therapy, combination therapy was best able to overcome resistance and bring about cell death. Doustvandi et al. [175] demonstrated that a combination therapy of zinc phthalocyanine PDT and doxorubicin chemotherapy had several interesting effects of melanoma cells in vitro. Not only did the combination therapy display a compounded effect leading to a significant decrease in cell viability, but pre-treatment with PDT at a low light dose sensitised the cancer cells to doxorubicin, allowing for lower chemotherapy doses to illicit comparable responses to those seen with high doxorubicin concentration monotherapy. Another study by Hwang et al. [176] demonstrated that a combination of photodynamic therapy using pheophorbide A together with a flagellin-adjuvanted cancer vaccine acted on the PD-1 pathway, well-known to promote melanoma immune evasion, to promote melanoma suppression by inducing an anti-tumour immune response in mice. Recent research suggests that combination therapies may be the way forward, offering synergetic effects to combat malignant and metastatic melanoma [177]. It has been suggested that the abscopal effect, which has been frequently reported in radiotherapy [178], might too play a role in PDT [179,180,181]. Ultimately, PDT, when combined with other treatments such as chemotherapy, immune checkpoint blockades or cancer vaccinations, might be best able to capitalise on these immunogenic effects and synergetically offer the best chance of eliminating metastatic melanoma.

## 8. Conclusions

We have recently made great advances regarding our understanding of cancer, resulting in the development of many next-generations therapies, including immunotherapies and targeted therapies. However, recent clinical trials have often been unable to detect major differences in treatment outcomes. In addition, the rate of new FDA drug approval is low. Our ability to eliminate cancer cells remains inadequate, and many drugs provide non-specific cytotoxicity resulting in side effects. This is considered to be due to the melanoma cells’ advanced capabilities of resisting the apoptotic signals initiated by treatment, thus allowing them to evade cell death. While PDT has been popularised and is reportedly promising for melanoma treatment, there are limitations to its clinical applicability, and we might be able to further enhance its efficacy using several measure. Antibody-mediated nanoparticles offer a sophisticated drug delivery system that could increase drug bioavailability and uptake and decrease off-target drug accumulation. By combining the three abovementioned treatment strategies and harnessing the anticancer benefits of each—the tolerability and controlled activation of PDT, the enhanced biodistribution and uptake of NPs and the specificity of immunotherapy—and additionally employing the use of SNAP-tag technology, it might be possible to enhance the current PDT techniques to achieve more efficacious destruction of melanoma cells via a targeted NP delivery system for PDT with reduced off-target toxicity.

## Figures and Tables

**Table 1:** List of approved photosensitisers for cancer management [32,33,34].

Photosensitiser	Cancer	Country/Region of Approval	Year of First Approval
Photofrin	Bladder cancer, lung cancer, advanced obstructive oesophageal cancer, early-stage non-small-cell lung cancer, cervical cancer	Canada, Japan, USA, Europe	1993
Foscan	Advanced head and neck squamous cell carcinoma	Europe	2001
Talaporfin sodium/Laserphyrin	Early-stage lung cancer	Japan	2004
5-ALA Ameluz/Levulan Metvix/Metvixia	Basal cell carcinoma, optical imaging in high-grade gliomas and bladder cancer	USA, Europe, New Zealand	2007
Redaporfin	Biliary tract cancer	Europe, USA	2015
SGX301 (synthetic hypericin)	Early stage cutaneous T-cell lymphoma	USA	2021
NIR-PIT (IR700) with Erbitux anti-EGFR mAb	Recurrent head and neck cancer	Japan	2021

**Table 2:** List of photosensitisers under investigation for melanoma PDT (2018 to 2022).

Study (Authors, Year)	Photosensitiser	Absorption Wavelength (nm)	Antibody	Nanoparticle
Tang et al., 2018 [165]	Indocyanine green (ICG)	808	None	Carboxylated poly (amido-amine)
Bazylińska et al., 2018 [166]	Chlorin e6 (Ce6)	600–630	None	Cubosomes
Bazylińska et al., 2018 [166]	Meso-tetraphenylporphine-Mn (III) chloride (TPP-Mn (III)cl)	530–570	None	Cubosomes
Clemente et al., 2019 [167]	Verteporfin (Ver)	690	None	Mesoporous silica nanoparticles (MSNs)
Lee et al., 2019 [168]	ICG	785	None	Chitosan-coated liposomes
Li et al., 2020 [169]	Pyropheophorbide a (Ppa)	670	None	Amphiphilic micelles
Li et al., 2020 [170]	Ce6	980	None	Mesoporous coated upconverting nanoparticles
Naidoo et al., 2019 [103]	Zinc phthalocyanine tetra-sulphonic acid	673	Melanoma inhibitory activity antigen	Gold
Ghazaeian et al., 2021 [171]	Curcumin	465	None	Silica
Biteghe et al., 2020 [14]	Hypericin with doxorubicin	561	None	None
Zhang et al., 2019 [30]	5-ALA with 5-FU and 5-MA pre-treatment	633 ± 10	None	None

## References

[1] Dratkiewicz E., Simiczyjew A., Mazurkiewicz J., Ziętek M., Matkowski R., Nowak D. (2021). Hypoxia and Extracellular Acidification as Drivers of Melanoma Progression and Drug Resistance. Cells.

[2] Bertolotto C. (2013). Melanoma: From melanocyte to genetic alterations and clinical options. Scientifica.

[3] Rutkowski P., Zdzienicki M., Nowecki Z.I., Van Akkooi A.C. (2010). Surgery of primary melanomas. Cancers.

[4] Gutiérrez-Castañeda L.D., Nova J.A., Tovar-Parra J.D. (2020). Frequency of mutations in BRAF, NRAS, and KIT in different populations and histological subtypes of melanoma: A systemic review. Melanoma Res..

[5] Sullivan R.J., Flaherty K. (2013). MAP kinase signaling and inhibition in melanoma. Oncogene.

[6] Amaral T., Sinnberg T., Meier F., Krepler C., Levesque M., Niessner H., Garbe C. (2017). The mitogen-activated protein kinase pathway in melanoma part I—Activation and primary resistance mechanisms to BRAF inhibition. Eur. J. Cancer.

[7] Takeda T., Tsubaki M., Sakamoto K., Ichimura E., Enomoto A., Suzuki Y., Itoh T., Imano M., Tanabe G., Muraoka O. (2016). Mangiferin, a novel nuclear factor kappa B-inducing kinase inhibitor, suppresses metastasis and tumor growth in a mouse metastatic melanoma model. Toxicol. Appl. Pharmacol..

[8] Madonna G., Ullman C.D., Gentilcore G., Palmieri G., Ascierto P.A. (2012). NF-κB as potential target in the treatment of melanoma. J. Transl. Med..

[9] Ambrosini G., Do C., Tycko B., Realubit R.B., Karan C., Musi E., Carvajal R.D., Chua V., Aplin A.E., Schwartz G.K. (2019). Inhibition of NF-κB-dependent signaling enhances sensitivity and overcomes resistance to bet inhibition in uveal melanoma. Cancer Res..

[10] Takeda T., Tsubaki M., Asano R., Itoh T., Imano M., Satou T., Nishida S. (2020). Dimethyl fumarate suppresses metastasis and growth of melanoma cells by inhibiting the nuclear translocation of NF-κB. J. Dermatol. Sci..

[11] Michaelis M., Rothweiler F., Nerreter T., van Rikxoort M., Zehner R., Dirks W.G., Wiese M., Cinatl J. (2014). Association between acquired resistance to PLX4032 (vemurafenib) and ATP-binding cassette transporter expression. BMC Res. Notes.

[12] Si X., Gao Z., Xu F., Zheng Y. (2020). SOX2 upregulates side population cells and enhances their chemoresistant ability by transactivating ABCC1 expression contributing to intrinsic resistance to paclitaxel in melanoma. Mol. Carcinog..

[13] Basu R., Qian Y., Mathes S., Terry J., Arnett N., Riddell T., Stevens A., Funk K., Bell S., Bokal Z. (2022). Growth hormone receptor antagonism downregulates ATP-binding cassette transporters contributing to improved drug efficacy against melanoma and hepatocarcinoma in vivo. Front. Oncol..

[14] Biteghe F.A.N., Padayachee E., Davids L.M., Chalomie N.E.T., Ndong J.C., Barth S. (2020). Desensitization of metastatic melanoma cells to therapeutic treatment through repeated exposure to dacarbazine. J. Photochem. Photobiol. B.

[15] Obrador E., Liu-Smith F., Dellinger R.W., Salvador R., Meysken F.L., Estrela J.M. (2019). Oxidative stress and antioxidants in the pathophysiology of malignant melanoma. Biol. Chem..

[16] Hansda S., Ghosh R. (2021). Bystander effect of ultraviolet A radiation protects A375 melanoma cells by induction of antioxidant defense. J. Environ. Sci. Health C Toxicol. Carcinog..

[17] Blázquez-Castro A., Stockert J.C. (2021). Biomedical overview of melanin. 1. Updating melanin biology and chemistry, physico-chemical properties, melanoma tumors, and photothermal therapy. Biocell.

[18] Melanoma: Statistics|Cancer.Net. https://www.cancer.net/cancer-types/melanoma/statistics.

[19] Dąbrowski J.M. (2017). Reactive Oxygen Species in Photodynamic Therapy: Mechanisms of Their Generation and Potentiation. Adv. Inorg. Chem..

[20] Sharma K.V., Bowers N., Davids L.M. (2011). Photodynamic therapy-induced killing is enhanced in depigmented metastatic melanoma cells. Cell Biol. Int..

[21] Davids L.M., Kleemann B., Cooper S., Kidson S.H. (2009). Melanomas display increased cytoprotection to hypericin-mediated cytotoxicity through the induction of autophagy. Cell Biol. Int..

[22] Turubanova V.D., Balalaeva I.V., Mishchenko T.A., Catanzaro E., Alzeibak R., Peskova N.N., Efimova I., Bachert C., Mitroshina E.V., Krysko O. (2019). Immunogenic cell death induced by a new photodynamic therapy based on photosens and photodithazine. J. ImmunoTher. Cancer.

[23] Naidoo C., Kruger C.A., Abrahamse H. (2019). Simultaneous photodiagnosis and photodynamic treatment of metastatic melanoma. Molecules.

[24] Correia J.H., Rodrigues J.A., Pimenta S., Dong T., Yang Z. (2021). Photodynamic Therapy Review: Principles, Photosensitizers, Applications, and Future Directions. Pharmaceutics.

[25] Luksiene Z. (2003). Photodynamic therapy: Mechanism of action and ways to improve the efficiency of treatment. Medicina.

[26] dos Santos A.F., de Almeida D.R.Q., Terra L.F., Baptista M.S., Labriola L. (2019). Photodynamic therapy in cancer treatment—An update review. J. Cancer Metastasis Treat..

[27] Kwiatkowski S., Knap B., Przystupski D., Saczko J., Kędzierska E., Knap-Czop K., Kotlińska J., Michel O., Kotowski K., Kulbacka J. (2018). Photodynamic therapy—Mechanisms, photosensitizers and combinations. Biomed. Pharmacother..

[28] Agostinis P., Berg K., Cengel K.A., Foster T.H., Girotti A.W., Gollnick S.O., Hahn S.M., Hamblin M.R., Juzeniene A., Kessel D. (2011). Photodynamic therapy of cancer: An update. CA Cancer J. Clin..

[29] Gilaberte Y., Milla L., Salazar N., Vera-Alvarez J., Kourani O., Damian A., Rivarola V., Roca M.J., Espada J., González S. (2014). Cellular intrinsic factors involved in the resistance of squamous cell carcinoma to photodynamic therapy. J. Investig. Dermatol..

[30] Zhang L., Ji Z., Zhang J., Yang S. (2019). Photodynamic therapy enhances skin cancer chemotherapy effects through autophagy regulation. Photodiagnosis Photodyn. Ther..

[31] Photodynamic Therapy to Treat Cancer. https://www.cancer.gov/about-cancer/treatment/types/photodynamic-therapy.

[32] Niculescu A.G., Mihai Grumezescu A., Photodynamic A. (2021). Photodynamic Therapy-An Up-to-Date Review. Appl. Sci..

[33] Gunaydin G., Gedik M.E., Ayan S. (2021). Photodynamic Therapy for the Treatment and Diagnosis of Cancer–A Review of the Current Clinical Status. Front. Chem..

[34] Hamblin M.R. (2020). Photodynamic Therapy for Cancer: What’s Past is Prologue. Photochem. Photobiol..

[35] Hadjur C., Richard M.J., Parat M.O., Jardon P., Favier A. (1996). Photodynamic effects of hypericin on lipid peroxidation and antioxidant status in melanoma cells. Photochem. Photobiol..

[36] Kleemann B., Loos B., Scriba T.J., Lang D., Davids L.M. (2014). St John’s Wort (*Hypericum perforatum* L.) photomedicine: Hypericin-photodynamic therapy induces metastatic melanoma cell death. PLoS ONE.

[37] Davids L.M., Kleemann B., Kacerovská D., Pizinger K., Kidson S.H. (2008). Hypericin phototoxicity induces different modes of cell death in melanoma and human skin cells. J. Photochem. Photobiol. B Biol..

[38] Yang J.F., Liu Y.R., Huang C.C., Ueng Y.F. (2018). The time-dependent effects of St John’s wort on cytochrome P450, uridine diphosphate-glucuronosyltransferase, glutathione S-transferase, and NAD(P)H-quinone oxidoreductase in mice. J. Food Drug Anal..

[39] Nicolussi S., Drewe J., Butterweck V., Meyer zu Schwabedissen H.E. (2020). Clinical relevance of St. John’s wort drug interactions revisited. Bri. J. Pharmacol..

[40] Scholz I., Liakoni E., Hammann F., Grafinger K.E., Duthaler U., Nagler M., Krähenbühl S., Haschke M. (2021). Effects of Hypericum perforatum (St John’s wort) on the pharmacokinetics and pharmacodynamics of rivaroxaban in humans. Br. J. Clin. Pharmacol..

[41] Colebatch A.J., Scolyer R.A. (2018). Trajectories of premalignancy during the journey from melanocyte to melanoma. Pathology.

[42] Faber D.J., Mik E.G., Aalders M.C.G., van Leeuwen T.G. (2003). Light absorption of (oxy-)hemoglobin assessed by spectroscopic optical coherence tomography. Opt. Lett..

[43] Hong G., Antaris A.L., Dai H. (2017). Near-infrared fluorophores for biomedical imaging. Nat. Biomed. Eng..

[44] Cao Q., Zhegalova N.G., Wang S.T., Akers W.J., Berezin M.Y. (2022). Multispectral imaging in the extended near-infrared window based on endogenous chromophores. J. Biomed. Opt..

[45] Cheng-Che L.E. (2019). Effects and interactions of increased environmental temperature and UV radiation on photoageing and photocarcinogenesis of the skin. Exp. Dermatol..

[46] Yanine Neira Z., Dicker Jiménez V., Ortegón Pulido L.F., Rueda Rugeles A.J., Buitrago-Medina D.A. (2022). Photoaging factors in patients from two healthcare centers in Colombia. J. Cosmet. Dermatol..

[47] Ash C., Dubec M., Donne K., Bashford T. (2017). Effect of wavelength and beam width on penetration in light-tissue interaction using computational methods. Lasers Med. Sci.

[48] Sordillo L.A., Pratavieira S., Pu Y., Salas-Ramirez K., Shi L., Zhang L., Budansky Y., Alfano R.R. (2014). Third therapeutic spectral window for deep tissue imaging. Proc. SPIE.

[49] Qian X., Peng X.-H., Ansari D.O., Yin-Goen Q., Chen G.Z., Shin D.M., Yang L., Young A.N., Wang M.D., Nie S. (2008). In vivo tumor targeting and spectroscopic detection with surface-enhanced Raman nanoparticle tags. Nat. Biotechnol..

[50] Lane L.A., Xue R., Nie S. (2018). Emergence of two near.r.r-infrared windows for in vivo and intraoperative SERS. Curr. Opin. Chem. Biol..

[51] Nagaya T., Nakamura Y., Sato K., Harada T., Choyke P.L., Hodge J.W., Schlom J., Kobayashi H. (2017). Near infrared photoimmunotherapy with avelumab, an anti-programmed death-ligand 1 (PD-L1) antibody. Oncotarget.

[52] Nagaya T., Nakamura Y., Sato K., Zhang Y.-F., Ni M., Choyke P.L., Ho M., Kobayashi H. (2016). Near infrared photoimmunotherapy with an anti-mesothelin antibody. Oncotarget.

[53] Mitsunaga M., Nakajima T., Sano K., Choyke P.L., Kobayashi H. (2012). Near Infrared Theranostic Photoimmunotherapy (PIT): Repeated Exposure of Light Enhances the Effect of Immunoconjugate. Bioconjug. Chem..

[54] Mitsunaga M., Ogawa M., Kosaka N., Rosenblum L.T., Choyke P.L., Kobayashi H. (2011). Cancer Cell-Selective In Vivo Near Infrared Photoimmunotherapy Targeting Specific Membrane Molecules. Nat. Med..

[55] Cognetti D.M., Johnson J.M., Curry J.M., Kochuparambil S.T., McDonald D., Mott F., Fidler M.J., Stenson K., Vasan N.R., Razaq M.A. (2021). Phase 1/2a, open-label, multicenter study of RM-1929 photoimmunotherapy in patients with locoregional, recurrent head and neck squamous cell carcinoma. Head Neck.

[56] Saavedra R., Rocha L.B., Dąbrowski J.M., Arnaut L.G. (2014). Modulation of biodistribution, pharmacokinetics, and photosensitivity with the delivery vehicle of a bacteriochlorin photosensitizer for photodynamic therapy. ChemMedChem.

[57] Kruger C., Abrahamse H. (2018). Utilisation of Targeted Nanoparticle Photosensitiser Drug Delivery Systems for the Enhancement of Photodynamic Therapy. Molecules.

[58] Mohanraj V.J., Chen Y. (2006). Nanoparticles—A Review. Trop. J. Pharm. Res..

[59] Lim C.-K., Heo J., Shin S., Jeong K., Seo Y.H., Jang W.-D., Park C.R., Park S.Y., Kim S., Kwon I.C. (2013). Nanophotosensitizers toward advanced photodynamic therapy of Cancer. Cancer Lett..

[60] Master A., Livingston M., sen Gupta A. (2013). Photodynamic nanomedicine in the treatment of solid tumors: Perspectives and challenges. J. Control Release.

[61] Crous A., Abrahamse H. (2020). Effective gold nanoparticle-antibody-mediated drug delivery for photodynamic therapy of lung cancer stem cells. Int. J. Mol. Sci..

[62] Zolnik B.S., González-Fernández Á., Sadrieh N., Dobrovolskaia M.A. (2010). Minireview: Nanoparticles and the immune system. Endocrinology.

[63] Dobrovolskaia M.A., Shurin M., Shvedova A.A. (2015). Current understanding of interactions between nanoparticles and the immune system. Toxicol. Appl. Pharmacol..

[64] Sharma J.N., Pattadar D.K., Mainali B.P., Zamborini F.P. (2018). Size Determination of Metal Nanoparticles Based on Electrochemically Measured Surface-Area-to-Volume Ratios. Anal. Chem..

[65] Liu Y., Yang G., Jin S., Xu L., Zhao C.X. (2020). Development of High-Drug-Loading Nanoparticles. ChemPlusChem.

[66] Sztandera K., Gorzkiewicz M., Klajnert-Maculewicz B. (2020). Nanocarriers in photodynamic therapy—in vitro and in vivo studies. Wiley Interdiscip. Rev. Nanomed. Nanobiotechnol..

[67] Nicol J.R., Dixon D., Coulter J.A. (2015). Gold nanoparticle surface functionalization: A necessary requirement in the development of novel nanotherapeutics. Nanomedicine.

[68] Mfouo-Tynga I., El-Hussein A., Abdel-Harith M., Abrahamse H. (2014). Photodynamic ability of silver nanoparticles in inducing cytotoxic effects in breast and lung cancer cell lines. Int. J. Nanomed..

[69] Kumar S.S.D., Houreld N.N., Kroukamp E.M., Abrahamse H. (2018). Cellular imaging and bactericidal mechanism of green-synthesized silver nanoparticles against human pathogenic bacteria. J. Photochem. Photobiol. B.

[70] Kumar S.S.D., Rajendran N.K., Houreld N.N., Abrahamse H. (2018). Recent advances on silver nanoparticle and biopolymer-based biomaterials for wound healing applications. Int. J. Biol. Macromol..

[71] Pradeep Chandran C., Mani Rahulan K., Ganesan S. Synthesis and study of photodynamic activity of silver nanoparticles. Proceedings of the Photonics 2010: 10th International Conference on Fiber Optics & Photonics.

[72] Erdogan O., Abbak M., Demirbolat G.M., Birtekocak F., Aksel M., Pasa S., Cevik O. (2019). Green synthesis of silver nanoparticles via Cynara scolymus leaf extracts: The characterization, anticancer potential with photodynamic therapy in MCF7 cells. PLoS ONE.

[73] Srinivasan S., Bhardwaj V., Nagasetti A., Fernandez-Fernandez A., McGoron A.J. (2016). Multifunctional surface-enhanced raman spectroscopy-detectable silver nanoparticles for combined photodynamic therapy and pH-triggered chemotherapy. J. Biomed. Nanotechnol..

[74] Aiello M.B.R., Castrogiovanni D., Parisi J., Azcárate J.C., Einschlag F.S.G., Gensch T., Bosio G.N., Mártire D. (2018). Photodynamic Therapy in HeLa Cells Incubated with Riboflavin and Pectin-coated Silver Nanoparticles. Photochem. Photobiol..

[75] Mahajan P.G., Dige N.C., Vanjare B.D., Eo S.-H., Seo S.-Y., Kim S.J., Hong S.-K., Choi C.-S., Lee K.H. (2019). A potential mediator for photodynamic therapy based on silver nanoparticles functionalized with porphyrin. J. Photochem. Photobiol. A Chem..

[76] Liu R., Yang Z., Zhang L., Zhao J., Hou C., Zhao S. (2020). A near infrared dye-coated silver nanoparticle/carbon dot nanocomposite for targeted tumor imaging and enhanced photodynamic therapy. Nanoscale Adv..

[77] Aghajanzadeh M., Zamani M., Kouchi F.R., Eixenberger J., Shirini D., Estrada D., Shirini F. (2022). Synergic Antitumor Effect of Photodynamic Therapy and Chemotherapy Mediated by Nano Drug Delivery Systems. Pharmaceutic.

[78] El-Hussein A., Mfouo-Tynga I., Abdel-Harith M., Abrahamse H. (2015). Comparative study between the photodynamic ability of gold and silver nanoparticles in mediating cell death in breast and lung cancer cell lines. J. Photochem. Photobiol. B.

[79] García Calavia P., Bruce G., Pérez-García L., Russell D.A. (2018). Photosensitiser-gold nanoparticle conjugates for photodynamic therapy of cancer. Photochem. Photobiol. Sci..

[80] Caro C.M.P., Klippstein R., Pozo D.P.A. (2010). Silver Nanoparticles: Sensing and Imaging Applications. Silver Nanoparticles.

[81] Guerrero A.R., Hassan N., Escobar C.A., Albericio F., Kogan M.J., Araya E. (2014). Gold nanoparticles for photothermally controlled drug release. Nanomedicine.

[82] Di Corato R., Palumberi D., Marotta R., Scotto M., Carregal-Romero S., Gil P.R., Parak W.J., Pellegrino T. (2012). Magnetic nanobeads decorated with silver nanoparticles as cytotoxic agents and photothermal probes. Small.

[83] El-Hussein A. (2016). Study DNA Damage after Photodynamic Therapy Using Silver Nanoparticles with A549 Cell Line. J. Mol. Nanotechnol. Nanomed..

[84] Emens L. (2012). Breast cancer immunobiology driving immunotherapy: Vaccines and immune checkpoint blockade. Expert Rev. Anticancer. Ther..

[85] Pettinato M.C. (2021). Introduction to Antibody-Drug Conjugates. Antibodies.

[86] Zahavi D., AlDeghaither D., O’Connell A., Weiner L.M. (2018). Enhancing antibody-dependent cell-mediated cytotoxicity: A strategy for improving antibody-based immunotherapy. Antib. Ther..

[87] Wang B., Yang C., Jin X., Du Q., Wu H., Dall’Acqua W., Mazor Y. (2020). Regulation of antibody-mediated complement-dependent cytotoxicity by modulating the intrinsic affinity and binding valency of IgG for target antigen. MAbs.

[88] Chen D.S., Mellman I.J.N. (2017). Elements of cancer immunity and the cancer–immune set point. Nature.

[89] Juneja V.R., McGuire K.A., Manguso R.T., LaFleur M.W., Collins N., Haining W.N., Freeman G.J., Sharpe A.H. (2017). PD-L1 on tumor cells is sufficient for immune evasion in immunogenic tumors and inhibits CD8 T cell cytotoxicity. J. Exp. Med..

[90] Tauriello D.V.F., Sancho E., Batlle E. (2021). Overcoming TGFβ-mediated immune evasion in cancer. Nat. Rev. Cancer.

[91] Daassi D., Mahoney K.M., Freeman G.J. (2020). The importance of exosomal PDL1 in tumour immune evasion. Nat. Rev. Immunol..

[92] Restifo N.P., Smyth M.J., Snyder A. (2016). Acquired resistance to immunotherapy and future challenges. Nat. Rev. Cancer.

[93] Thery J.-C., Spano J.-P., Azria D., Raymond E., Penault Llorca F. (2014). Resistance to human epidermal growth factor receptor type 2-targeted therapies. Eur. J. Cancer.

[94] Shapiro C.L. (2016). Highlights of Recent Findings on Quality-of-Life Management for Patients with Cancer and Their Survivors. JAMA Oncol..

[95] Alley S.C., Okeley N.M., Senter P.D. (2010). Antibody–drug conjugates: Targeted drug delivery for cancer. Curr. Opin. Chem. Biol..

[96] Pohlit H., Bellinghausen I., Schoömer M., Heydenreich B.R., Saloga J., Frey H.J.B. (2015). Biodegradable pH-sensitive poly (ethylene glycol) nanocarriers for allergen encapsulation and controlled release. Biomacromolecules.

[97] von Felbert V., Bauerschlag D., Maass N., Bräutigam K., Meinhold-Heerlein I., Woitok M., Barth S., Hussain A.F. (2016). A specific photoimmunotheranostics agent to detect and eliminate skin cancer cells expressing EGFR. J. Cancer Res. Clin. Oncol..

[98] Leong A.S.Y., Zhuang Z. (2011). The Changing Role of Pathology in Breast Cancer Diagnosis and Treatment. Pathobiology.

[99] Panieri E. (2012). Breast cancer screening in developing countries. Best Pr. Res. Clin. Obstet. Gynaecol..

[100] Mohd-Zahid M.H., Mohamud R., Abdullah C.A.C., Lim J., Alem H., Hanaffi W.N.W., Iskandar Z.A. (2020). Colorectal cancer stem cells: A review of targeted drug delivery by gold nanoparticles. RSC Adv..

[101] Crous A., Abrahamse H. (2019). Photodynamic Therapy and Lung Cancer Stem Cells—The effects of AlPcS4Cl on Isolated Lung Cancer Stem Cells. Food Sci. Hum. Wellness.

[102] Ning S.-T., Lee S.-Y., Wei M.-F., Peng C.-L., Lin S.Y.-F., Tsai M.-H., Lee P.-C., Shih Y.-H., Lin C.-Y., Luo T.-Y. (2016). Targeting Colorectal Cancer Stem-Like Cells with Anti-CD133 Antibody-Conjugated SN-38 Nanoparticles. ACS Appl. Mater. Interfaces.

[103] Naidoo C., Kruger C.A., Abrahamse H. (2019). Targeted photodynamic therapy treatment of in vitro A375 metastatic melanoma cells. Oncotarget.

[104] Simelane N.W.N., Kruger C.A., Abrahamse H. (2021). Targeted nanoparticle photodynamic diagnosis and therapy of colorectal cancer. Int. J. Mol. Sci..

[105] Tai S.-P., Wu Y., Shieh D.-B., Chen L.-J., Lin K.-J., Yu C.-H., Chu S.-W., Chang C.-H., Shi X.-Y., Wen Y.-C. (2007). Molecular imaging of cancer cells using plasmon-resonant-enhanced third-harmonic-generation in silver nanoparticles. Adv. Mater..

[106] Khristunova Y., Korotkova E., Kratochvil B., Barek J., Dorozhko E., Vyskocil V., Plotnikov E., Voronova O., Sidelnikov V. (2019). Preparation and Investigation of Silver Nanoparticle-Antibody Bioconjugates for Electrochemical Immunoassay of Tick-Borne Encephalitis. Sensors.

[107] Szymanski M.S., Porter R.A. (2013). Preparation and quality control of silver nanoparticle-antibody conjugate for use in electrochemical immunoassays. J. Immunol. Methods.

[108] Pollok N.E., Rabin C., Smith L., Crooks R.M. (2019). Orientation-Controlled Bioconjugation of Antibodies to Silver Nanoparticles. Conjug. Chem.

[109] Nima Z.A., Alwbari A.M., Dantuluri V., Hamzah R.N., Sra N., Motwani P., Arnaoutakis K., Levy R.A., Bohliqa A.F., Nedosekin D. (2017). Targeting nano drug delivery to cancer cells using tunable, multi-layer, silver-decorated gold nanorods. J. Appl. Toxicol..

[110] Bösmüller H., Fischer A., Pham D.L., Fehm T., Capper D., von Deimling A., Bonzheim I., Staebler A., Fend F. (2013). Detection of the BRAF V600E mutation in serous ovarian tumors: A comparative analysis of immunohistochemistry with a mutation-specific monoclonal antibody and allele-specific PCR. Hum. Pathol..

[111] Fitting J., Blume T., Ten Haaf A., Blau W., Gattenlöhner S., Tur M.K., Barth S. (2015). Phage display-based generation of novel internalizing antibody fragments for immunotoxin-based treatment of acute myeloid leukemia. MAbs.

[112] Axup J.Y., Bajjuri K.M., Ritland M., Hutchins B.M., Kim C.H., Kazane S.A., Halder R., Forsyth J.S., Santidrian A.F., Stafin K. (2012). Synthesis of site-specific antibody-drug conjugates using unnatural amino acids. Proc. Natl. Acad. Sci. USA.

[113] Liu J., Dang H., Wang X.W. (2018). The significance of intertumor and intratumor heterogeneity in liver cancer. Exp. Mol. Med..

[114] Robert C., Schachter J., Long G.V., Arance A., Grob J.J., Mortier L., Daud A., Carlino M.S., McNeil C., Lotem M. (2015). Pembrolizumab versus ipilimumab in advanced melanoma. N. Engl. J. Med..

[115] Robert C., Long G.V., Brady B., Dutriaux C., Maio M., Mortier L., Hassel J.C., Rutkowski P., McNeil C., Kalinka-Warzocha E. (2015). Nivolumab in previously untreated melanoma without BRAF mutation. N. Engl. J. Med..

[116] Tawbi H.A., Schadendorf D., Lipson E.J., Ascierto P.A., Matamala L., Gutiérrez E.C., Rutkowski P., Gogas H.J., Lao C.D., De Menezes J.J. (2022). Relatlimab and Nivolumab versus Nivolumab in Untreated Advanced Melanoma. N. Engl. J. Med..

[117] Paik J. (2022). Nivolumab Plus Relatlimab: First Approval. Drugs.

[118] Milton Harris J., Chess R.B. (2003). Effect of pegylation on pharmaceuticals. Nat. Rev. Drug Discov..

[119] Hamblett K.J., Senter P.D., Chace D.F., Sun M.M.C., Lenox J., Cerveny C.G., Kissler K.M., Bernhardt S.X., Kopcha A.K., Zabinski R.F. (2004). Effects of Drug Loading on the Antitumor Activity of a Monoclonal Antibody Drug Conjugate. Proc. Am. Assoc. Cancer Res..

[120] Lyon R.P., Bovee T.D., Doronina S.O., Burke P.J., Hunter J.H., Neff-LaFord H.D., Jonas M., Anderson M.E., Setter J.R., Senter P.D. (2015). Reducing hydrophobicity of homogeneous antibody-drug conjugates improves pharmacokinetics and therapeutic index. Nat. Biotechnol..

[121] Simmons J.K., Burke P.J., Cochran J.H., Pittman P.G., Lyon R.P. (2020). Reducing the antigen-independent toxicity of antibody-drug conjugates by minimizing their non-specific clearance through PEGylation. Toxicol. Appl. Pharmacol..

[122] Oliveira S., Van Dongen G.A.M.S., Stigter-Van Walsum M., Roovers R.C., Stam J.C., Mali W., Van Diest P.J., Van Bergen En Henegouwen P.M.P. (2012). Rapid Visualization of Human Tumor Xenografts through Optical Imaging with a Near-infrared Fluorescent Anti—Epidermal Growth Factor Receptor Nanobody. Mol. Imaging.

[123] Zangemeister-Wittke U. (2005). Antibodies for targeted cancer therapy–technical aspects and clinical perspectives. Pathobiology.

[124] Dolan M.E., Moschel R.C., Pegg A.E. (1990). Depletion of mammalian O6-alkylguanine-DNA alkyltransferase activity by O6-benzylguanine provides a means to evaluate the role of this protein in protection against carcinogenic and therapeutic alkylating agents. Proc. Natl. Acad. Sci. USA.

[125] Keppler A., Kindermann M., Gendreizig S., Pick H., Vogel H., Johnsson K. (2004). Labeling of fusion proteins of O6-alkylguanine-DNA alkyltransferase with small molecules in vivo and in vitro. Methods.

[126] Pegg A.E., Dolan M.E., Moschel R.C. (1995). Structure, function, and inhibition of O6-alkylguanine-DNA alkyltransferase. Prog. Nucleic Acid. Res. Mol. Biol..

[127] Kampmeier F., Ribbert M., Nachreiner T., Dembski S., Beaufils F., Brecht A., Barth S. (2009). Site-specific, covalent labeling of recombinant antibody fragments via fusion to an engineered version of 6-O-alkylguanine DNA alkyltransferase. Bioconjug. Chem..

[128] Harding F.A., Stickler M.M., Razo J., DuBridge R. (2010). The immunogenicity of humanized and fully human antibodies: Residual immunogenicity resides in the CDR regions. MAbs.

[129] Holliger P., Hudson P.J. (2005). Engineered antibody fragments and the rise of single domains. Nat. Biotechnol..

[130] Rodrigo G., Gruvegård M., van Alstine J.M. (2015). Antibody Fragments and Their Purification by Protein L Affinity Chromatography. Antibodies.

[131] Puettmann C., Kolberg K., Hagen S., Schmies S., Fischer R., Naehring J., Barth S. (2013). A monoclonal antibody for the detection of SNAP/CLIP-tagged proteins. Immunol. Lett..

[132] Kampmeier F., Niesen J., Koers A., Ribbert M., Brecht A., Fischer R., Kießling F., Barth S., Thepen T. (2010). Rapid optical imaging of EGF receptor expression with a single-chain antibody SNAP-tag fusion protein. Eur. J. Nucl. Med. Mol. Imaging.

[133] Kijanka M., Warnders F.J., El Khattabi M., Lub-de Hooge M., van Dam G.M., Ntziachristos V., de Vries L., Oliveira S., van Bergen En Henegouwen P.M. (2013). Rapid optical imaging of human breast tumour xenografts using anti-HER2 VHHs site-directly conjugated to IRDye 800CW for image-guided surgery. Eur. J. Nucl. Med. Mol. Imaging.

[134] Gong H., Kovar J.L., Baker B., Zhang A., Cheung L., Draney D.R., Corrêa I.R., Xu M.Q., Olive D.M. (2012). Near-Infrared Fluorescence Imaging of Mammalian Cells and Xenograft Tumors with SNAP-Tag. PLoS ONE.

[135] Li Q., White J.B., Peterson N.C., Rickert K.W., Lloyd C.O., Allen K.L., Rosenthal K., Gao X., Wu H., Dall’Acqua W.F. (2018). Tumor uptake of pegylated diabodies: Balancing systemic clearance and vascular transport. Journal of Controlled Release. J. Control. Release.

[136] Pan H., Liu J., Deng W., Xing J., Li Q., Wang Z. (2018). Site-specific PEGylation of an anti-CEA/CD3 bispecific antibody improves its antitumor efficacy. Int. J. Nanomed..

[137] Hussain A.F., Kampmeier F., von Felbert V., Merk H.-F., Tur M.K., Barth S. (2011). SNAP-Tag Technology Mediates Site Specific Conjugation of Antibody Fragments with a Photosensitizer and Improves Target Specific Phototoxicity in Tumor Cells. Bioconjug. Chem..

[138] Bauerschlag D., Meinhold-Heerlein I., Maass N., Bleilevens A., Bräutigam K., Al Rawashdeh W., Di Fiore S., Haugg A.M., Gremse F., Steitz J. (2017). Detection and Specific Elimination of EGFR + Ovarian Cancer Cells Using a Near Infrared Photoimmunotheranostic Approach. Pharm. Res..

[139] Davids L.M., Biteghe F.N., Padayachee E., Barth S. (2019). Targeted photodynamic therapy enhances the therapeutic efficacy of combination therapy (PDT and chemotherapy) on chemoresistant melanoma cells. Cancer Res..

[140] Hussain A.F., Heppenstall P.A., Kampmeier F., Meinhold-Heerlein I., Barth S. (2019). One-step site-specific antibody fragment auto-conjugation using SNAP-tag technology. Nat. Protoc..

[141] Ritchie M., Tchistiakova L., Scott N. (2013). Implications of receptor-mediated endocytosis and intracellular trafficking dynamics in the development of antibody drug conjugates. MAbs.

[142] Hamid O., Robert C., Daud A., Hodi F.S., Hwu W.J., Kefford R., Wolchok J.D., Hersey P., Joseph R., Weber J.S. (2019). Five-year survival outcomes for patients with advanced melanoma treated with pembrolizumab in KEYNOTE-001. Ann. Oncol..

[143] Almutairi A.R., McBride A., Slack M., Erstad B.L., Abraham I. (2020). Potential Immune-Related Adverse Events Associated with Monotherapy and Combination Therapy of Ipilimumab, Nivolumab, and Pembrolizumab for Advanced Melanoma: A Systematic Review and Meta-Analysis. Front. Oncol..

[144] Muenst S., Schaerli A.R., Gao F., Däster S., Trella E., Droeser R.A., Muraro M.G., Zajac P., Zanetti R., Gillanders W.E. (2014). Expression of programmed death ligand 1 (PD-L1) is associated with poor prognosis in human breast cancer. Breast Cancer Res. Treat..

[145] Rose A.A.N., Armstrong S.M., Hogg D., Butler M.O., Saibil S.D., Arteaga D.P., Muniz T.P., Kelly D., Ghazarian D., King I. (2021). Biologic subtypes of melanoma predict survival benefit of combination anti-PD1+anti-CTLA4 immune checkpoint inhibitors versus anti-PD1 monotherapy. J. Immunother. Cancer.

[146] Curran M.A., Montalvo W., Yagita H., Allison J.P. (2010). PD-1 and CTLA-4 combination blockade expands infiltrating T cells and reduces regulatory T and myeloid cells within B16 melanoma tumors. Proc. Natl. Acad. Sci. USA.

[147] Kim Y.J., Won C.H., Lee M.W., Choi J.H., Chang S.E., Lee W.J. (2020). Correlation Between Tumor-Associated Macrophage and Immune Checkpoint Molecule Expression and Its Prognostic Significance in Cutaneous Melanoma. J. Clin. Med..

[148] Tong J.T.W., Harris P.W.R., Brimble M.A., Kavianinia I. (2021). An Insight into FDA Approved Antibody-Drug Conjugates for Cancer Therapy. Molecules.

[149] Hoffmann R.M., Crescioli S., Mele S., Sachouli E., Cheung A., Chui C.K., Andriollo P., Jackson P.J.M., Lacy K.E., Spicer J.F. (2020). A Novel Antibody-Drug Conjugate (ADC) Delivering a DNA Mono-Alkylating Payload to Chondroitin Sulfate Proteoglycan (CSPG4)-Expressing Melanoma. Cancers.

[150] Price M.A., Wanshura L.E.C., Yang J., Carlson J., Xiang B., Li G., Ferrone S., Dudek A.Z., Turley E.A., McCarthy J.B. (2011). CSPG4, a potential therapeutic target, facilitates malignant progression of melanoma. Pigment. Cell Melanoma Res..

[151] Yang J., Price M.A., Neudauer C.L., Wilson C., Ferrone S., Xia H., Iida J., Simpson M.A., McCarthy J.B. (2004). Melanoma chondroitin sulfate proteoglycan enhances FAK and ERK activation by distinct mechanisms. J. Cell Biol..

[152] Svendsen A., Verhoeff J.J., Immervoll H., Brøgger J.C., Kmiecik J., Poli A., Netland I.A., Prestegarden L., Planaguma J., Torsvik A. (2011). Expression of the progenitor marker NG2/CSPG4 predicts poor survival and resistance to ionising radiation in glioblastoma. Acta Neuropathol..

[153] Uranowska K., Kalic T., Valtsanidis V., Kitzwögerer M., Breiteneder H., Hafner C. (2021). Expression of chondroitin sulfate proteoglycan 4 (CSPG4) in melanoma cells is downregulated upon inhibition of BRAF. Oncol. Rep..

[154] Ghosh A., Syed S., Kumar M., Carpenter T.J., Teixeira J.M., Houairia N., Negi S., Tanwar P.S. (2020). In Vivo Cell Fate Tracing Provides No Evidence for Mesenchymal to Epithelial Transition in Adult Fallopian Tube and Uterus. Cell Rep..

[155] Yang J., Price M.A., Li G.Y., Bar-Eli M., Salgia R., Jagedeeswaran R., Carlson J.H., Ferrone S., Turley E.A., McCarthy J.B. (2009). Melanoma proteoglycan modifies gene expression to stimulate tumor cell motility, growth, and epithelial-to-mesenchymal transition. Cancer Res..

[156] Wen Y., Makagiansar I.T., Fukushi J.-I., Liu F.-T., Fukuda M.N., Stallcup W.B. (2006). Molecular basis of interaction between NG2 proteoglycan and galectin-3. J. Cell. Biochem..

[157] Natali P., Bigotti A., Cavalieri R., Wakabayaski S., Taniguchi M., Ferrone S. (1985). Distribution of a cross-species melanoma-associated antigen in normal and neoplastic human tissues. J. Investig. Dermatol.

[158] Wang X., Osada T., Wang Y., Yu L., Sakakura K., Katayama A., McCarthy J.B., Brufsky A., Chivukula M., Khoury T. (2010). CSPG4 protein as a new target for the antibody-based immunotherapy of triple-negative breast cancer. Natl. Cancer Inst..

[159] Bonhoure A., Henry L., Morille M., Aissaoui N., Bellot G., Stoebner P.E., Vidal M. (2021). Melanotransferrin is efficiently sorted on the surface of exosomes secreted by melanoma cells. Melanoma Res..

[160] Abrams T., Connor A., Fanton C., Cohen S.B., Huber T., Miller K., Hong E.E., Niu X., Kline J., Ison-Dugenny M. (2018). Preclinical Antitumor Activity of a Novel Anti–c-KIT Antibody–Drug Conjugate against Mutant and Wild-type c-KIT–Positive Solid Tumors. Clin. Cancer Res..

[161] Cardillo T.M., Govindan S.V., Zalath M.B., Rossi D.L., Wang Y., Chang C.-H., Goldenberg D.M. (2018). IMMU-140, a novel SN-38 antibody-drug conjugate targeting HLA-DR, mediates dual cytotoxic effects in hematologic cancers and malignant melanoma. Mol. Cancer Ther..

[162] Capone E., Lamolinara A., D’Agostino D., Rossi C., De Laurenzi V., Iezzi M., Iacobelli S., Sala G. (2018). EV20-mediated delivery of cytotoxic auristatin MMAF exhibits potent therapeutic efficacy in cutaneous melanoma. J. Control. Release.

[163] Ott P.A., Pavlick A.C., Johnson D.B., Hart L.L., Infante J.R., Luke J.J., Lutzky J., Rothschild N.E., Spitler L.E., Cowey C.L. (2019). A phase 2 study of glembatumumab vedotin, an antibody-drug conjugate targeting glycoprotein NMB, in patients with advanced melanoma. Cancer.

[164] Boshuizen J., Koopman L.A., Krijgsman O., Shahrabi A., van den Heuvel E.G., Ligtenberg M.A.A., Vredevoogd D.W., Kemper K., Kuilman T., Song J.-Y. (2018). Cooperative targeting of melanoma heterogeneity with an AXL antibody-drug conjugate and BRAF/MEK inhibitors. Nat. Med..

[165] Tang J., Zhou H., Hou X., Wang L., Li Y., Pang Y., Chen C., Jiang G., Liu Y. (2018). Enhanced anti-tumor efficacy of temozolomide-loaded carboxylated poly(amido-amine) combined with photothermal/photodynamic therapy for melanoma treatment. Cancer Lett..

[166] Bazylińska U., Kulbacka J., Schmidt J., Talmon Y., Murgia S. (2018). Polymer-free cubosomes for simultaneous bioimaging and photodynamic action of photosensitizers in melanoma skin cancer cells. J. Colloid Interface Sci..

[167] Clemente N., Miletto I., Gianotti E., Invernizzi M., Marchese L., Dianzani U., Renò F. (2019). Verteporfin-loaded mesoporous silica nanoparticles inhibit mouse melanoma proliferation in vitro and in vivo. J. Photochem. Photobiol. B Biol..

[168] Lee E.H., Lim S.J., Lee M.K. (2019). Chitosan-coated liposomes to stabilize and enhance transdermal delivery of indocyanine green for photodynamic therapy of melanoma. Carbohydr. Polym..

[169] Li J., Xue Y., Tian J., Liu Z., Zhuang A., Gu P., Zhou H., Zhang W., Fan X. (2020). Fluorinated-functionalized hyaluronic acid nanoparticles for enhanced photodynamic therapy of ocular choroidal melanoma by ameliorating hypoxia. Carbohydr. Polym..

[170] Li D., Ren J., Li J., Zhang Y., Lou Y., Zhu J., Liu P., Chen Y., Yu Z., Zhao L. (2021). Ferroptosis-apoptosis combined anti-melanoma immunotherapy with a NIR-responsive upconverting mSiO2 photodynamic platform. Chem. Eng. J..

[171] Ghazaeian M., Khorsandi K., Hosseinzadeh R., Naderi A., Abrahamse H. (2021). Curcumin–silica nanocomplex preparation, hemoglobin and DNA interaction and photocytotoxicity against melanoma cancer cells. J. Biomol. Struct. Dyn..

[172] Omura G., Honma Y., Matsumoto Y., Shinozaki T., Itoyama M., Eguchi K., Sakai T., Yokoyama K., Watanabe T., Ohara A. (2022). Transnasal photoimmunotherapy with cetuximab sarotalocan sodium: Outcomes on the local recurrence of nasopharyngeal squamous cell carcinoma. Auris Nasus Larynx.

[173] DeWitt J.M., Sandrasegaran K., O’Neil B., House M.G., Zyromski N.J., Sehdev A., Perkins S.M., Flynn J., McCranor L., Shahda S. (2019). Phase 1 study of EUS-guided photodynamic therapy for locally advanced pancreatic cancer. Gastrointest. Endosc..

[174] Hanada Y., Pereira S.P., Pogue B., Maytin E.V., Hasan T., Linn B., Mangels-Dick T., Wang K.K. (2021). EUS-guided verteporfin photodynamic therapy for pancreatic cancer. Gastrointest. Endosc..

[175] Doustvandi M.A., Mohammadnejad F., Mansoori B., Tajalli H., Mohammadi A., Mokhtarzadeh A., Baghbani E., Khaze V., Hajiasgharzadeh K., Moghaddam M.M. (2019). Photodynamic therapy using zinc phthalocyanine with low dose of diode laser combined with doxorubicin is a synergistic combination therapy for human SK-MEL-3 melanoma cells. Photodiagnosis Photodyn. Ther..

[176] Hwang H.S., Cherukula K., Bang Y.J., Vijayan V., Moon M.J., Thiruppathi J., Puth S., Jeong Y.Y., Park I.-K., Lee S.E. (2020). Combination of Photodynamic Therapy and a Flagellin-Adjuvanted Cancer Vaccine Potentiated the Anti-PD-1-Mediated Melanoma Suppression. Cells.

[177] Khorsandi K., Hosseinzadeh R., Chamani E. (2020). Molecular interaction and cellular studies on combination photodynamic therapy with rutoside for melanoma A375 cancer cells: An in vitro study. Cancer Cell Int..

[178] Postow M.A., Callahan M.K., Barker C.A., Yamada Y., Yuan J., Kitano S., Mu Z., Rasalan T., Adamow M., Ritter E. (2012). Immunologic Correlates of the Abscopal Effect in a Patient with Melanoma. N. Engl. J. Med..

[179] Xie Q., Li Z., Liu Y., Zhang D., Su M., Niitsu H., Lu Y., Coffey R.J., Bai M. (2021). Translocator protein-targeted photodynamic therapy for direct and abscopal immunogenic cell death in colorectal cancer. Acta Biomater..

[180] Lou J., Aragaki M., Bernards N., Kinoshita T., Mo J., Motooka Y., Ishiwata T., Gregor A., Chee T., Chen Z. (2021). Repeated porphyrin lipoprotein-based photodynamic therapy controls distant disease in mouse mesothelioma via the abscopal effect. Nanophotonics.

[181] Ghosh P., Hanada Y., Linn B., Mangels-Dick T., Roy B., Wang K. (2021). Abscopal Effect of Intratumoral Photodynamic Therapy Is Associated with Increased Tumor Directed T Cells. Am. J. Gastroenterol..

